# Breastfeeding and Allergy Effect Modified by Genetic, Environmental, Dietary, and Immunological Factors

**DOI:** 10.3390/nu14153011

**Published:** 2022-07-22

**Authors:** Hanna Danielewicz

**Affiliations:** 1st Clinical Department of Pediatrics, Allergology and Cardiology, Wroclaw Medical University, ul. Chałubińskiego 2a, 50-368 Wrocław, Poland; hanna.danielewicz@umed.wroc.pl

**Keywords:** breastfeeding, maternal atopy, epigenetics, food allergy

## Abstract

Breastfeeding (BF) is the most natural mode of nutrition. Its beneficial effect has been revealed in terms of both the neonatal period and those of lifelong effects. However, as for protection against allergy, there is not enough data. In the current narrative review, the literature within the last five years from clinical trials and population-based studies on breastfeeding and allergy from different aspects was explored. The aim of this review was to explain how different factors could contribute to the overall effect of BF. Special consideration was given to accompanying exposure to cow milk, supplement use, the introduction of solid foods, microbiota changes, and the epigenetic function of BF. Those factors seem to be modifying the impact of BF. We also identified studies regarding BF in atopic mothers, with SCFA as a main player explaining differences according to this status. Conclusion: Based on the population-based studies, breastfeeding could be protective against some allergic phenotypes, but the results differ within different study groups. According to the new research in that matter, the effect of BF could be modified by different genetic (HMO composition), environmental (cesarean section, allergen exposure), dietary (SCFA, introduction of solid food), and immunologic factors (IgG, IgE), thus partially explaining the variance.

## 1. Introduction

Breastfeeding is the most natural mode of nutrition in the first months of life. Its beneficial effect has been elucidated, not only in the neonatal period but also in terms of lifelong impacts. Recent large meta-analyses on this style of feeding have revealed protection against metabolic conditions, such as overweight and diabetes, as well as against early childhood infections. However, no evidence, little evidence, or inconclusive results has been indicated for breastfeeding and different allergy phenotypes [1]. In earlier studies, some protective effect was visible for atopic dermatitis in infancy [2]. In the context of developing allergies early in life, two main types of interventions have been studied over the last few years. The first was exclusive breastfeeding in the first 4 to 6 months of life in comparison to formula feeding; the second was the early introduction of allergenic foods. The second aspect has emerged since the publication of the LEAP study results showing a protective effect of the early introduction of peanuts on the prevention of peanut allergy [3]. Nevertheless, this intervention did not affect in any way sensitization to other food allergens.

The LEAP study was one of the first studies investigating the early introduction of solid food, similar to the EAT study [4] that introduced egg, peanut, sesame, cod fish, and wheat [5], the STAR and HealthyNuts studies that introduced egg, and PreventADALL [6]. In spite of early promising results, some of these studies showed no protective effect [7].

The main mechanism discussed in the context of the protective performance of breast milk is epigenetic imprinting. This phenomenon could explain long-lasting health benefits. It has been speculated that breastfeeding impacts epigenetic processes both by the direct effect of bio-compounds present in human milk and indirect effects depending on the shaping of the microbiome in the neonatal gut and the related presence of bacteria metabolites such as butyrate and propionate, which operate as active compounds. The active bio-compounds in breast milk could work at different levels of epigenetic imprinting. These active elements are mainly dendritic cells containing live maternal gut bacteria, prostaglandin J and PUFA exhibiting metabolic effects, lactoferrin with its ability to bind bacterial CpG thus preventing the NFκB response against flora, microvesicles with the demonstrated effect of inhibiting atopic sensitization, fat globules containing microRNA, which target several infant genes, and multipotential stem cells [1,8].

Due to existing controversies on the subject regarding the protection of BF against allergy, we have searched the most recent literature through the two databases: PubMed and Embase, with the search term: “breastfeeding and allergy” for any new findings in the last 5 years, within the matter including clinical trials, randomized clinical trials, and population-based studies, with the respect of the sub-population of atopic mothers. We have included in this narrative review all the studies identified if they were related to BF and allergy or immune outcomes. The aim of this review was to explain how different factors could contribute to the overall effect of BF. The summary of the studies is presented in Table 1.

## 2. The Effect of Breastfeeding as an Only Exposure

Few studies have considered the protective effect of breastfeeding by itself, with emerging contraindicative results. Breastfeeding seemed to increase the risk of allergic rhinitis and allergic sensitization [11] and decrease the risk of asthma [18]. In the first study, the population born from 1937 to 1969 in the U.K. was examined with self-reported allergic outcomes. Since nowadays we observe an increase in allergies in the younger population (10–30 years), there is doubt as to whether we can extrapolate the results from the older group with different environmental exposures in the first years of life. As the authors concluded, the year of birth, socioeconomic status, and smoking status had high confounding power in the analysis, which confirms that lifestyle factors modify the effect of breastfeeding.

The second study showed an association between exclusive breastfeeding in the first 4 months of life and the composition of the intestinal metabolome at 3 years of age. This relation seemed to further mediate the association between protection against asthma and breastfeeding. From other possible predictors of a child’s intestinal metabolome, such as antibiotic use, cesarean section, having siblings, or dog ownership, only breastfeeding was an independent factor affecting the metabolome at 3 years.

Contrary to these results, the promotion of breastfeeding, i.e., prolonged duration and exclusivity for infants born in 1996–1997 in Belarus, has been shown to reduce the risk of flexural atopic dermatitis but had no impact on spirometry at 16 years [12]. Another large study in China has shown a protective effect against different allergic conditions. More than 10,000 children aged 6–11 years were evaluated for the reported diagnosis of asthma and other allergic conditions. Factors such as male sex, high socioeconomic status, cesarean section, being an only child, and a family history of allergy were associated with an increased risk for having asthma and other allergic conditions at that age, while prolonged (>6 months) breastfeeding was related to a decreased risk. In addition, breastfeeding attenuated the risk connected to other factors [14]. In GINI (German Infant Nutritional Intervention) study, full breastfeeding showed no effect on eczema and asthma, but a risk reduction for allergic rhinitis was observed [13].

These opposing results could be the effect of the complex nature of different exposures in the first years of life. Including all of them could bring some explanation, so if one is missing, controversies emerge.

## 3. Exposure to Cow Milk

One of the factors that could modify the effect of BF is cow milk exposure. Two contradictory study results have been published regarding early exposure to cow milk in breastfed infants. In the first ABC trial (Atopy induced by Breastfeeding or Cow’s milk formula), introducing milk formula at the earliest in the first 3 days of life was found to increase the risk of further allergy to not only cow milk but also other food allergens. In this trial, neonates received either breast milk and an amino acid formula as supplementation if necessary or breast milk and cow milk formula. Sensitization to cow milk and other secondary outcomes such as anaphylaxis and food allergy were estimated in the second year of life [9]. In the second study (SPADE—Strategy for Prevention of milk Allergy by Daily ingestion of infant formula in Early infancy), avoiding cow milk formula in comparison to feeding with at least 10 mL in the period between 1 and 2 months of life increased the risk of having an allergy, measured by OFC (oral food challenge) and sIgE and SPT (skin prick test) at the age of 6 months [10]. In both trials, infants in the cow milk avoidance arm received an alternative formula containing amino acids in the first case and soy formula in the second, so the results could have been affected by the impact of these formulas on the outcomes. The question is what was really studied: cow milk formula vs. soy/amino acid formula or avoiding cow milk allergens versus exposure. In addition, timing could make a huge difference here since the first days of life could be a very sensitive period for allergy development.

## 4. Breastfeeding and Changes in the Microbiota

Another factor that could both reflect and modify the effect of BF is microbiota composition. As changes in the gut microbiota are believed to be the main factor responsible for the immunomodulatory effect of breast milk, some studies have focused solely on this parameter. Surprisingly, breastfed children have shown lover diversity levels in comparison to formula-fed children. Despite having lower biodiversity, breastfed infants had more beneficial genera such as *Bifidobacterium* and *Lactobacillus*. This pattern appears to be beneficial according to the immaturity of the neonatal immune system. Apart from breastfeeding, ethnicity and maternal diet during pregnancy have some effect on stool microbiota at the age of 3–6 months, but not as strong as human milk [16]. A similar effect regarding microbiome diversity was confirmed in another study at different time points, i.e., 3, 6, 9, and 12 months of life [26]. In a large meta-analysis of seven microbiome studies, five cohorts, and 684 infants, the differences in the microbiome in relation to the mode of feeding were visible and persisted after 6 months of life (up to 2 years). Both diversity and the age of the microbiome were lower in breastfed infants in comparison to formula-fed infants. These differences were observed for composition and functional pathways, and the mode of delivery was a factor modifying the difference [27].

Children born by cesarean section can have more *Clostridium* in the gut microbiome [17]. *Clostridium* colonization is believed to be the main effector of harmful effects on different aspects of human health, as it has been revealed that it has an impact on microbiome composition, only in exclusively breast infants but not in formula-fed infants, suggesting an already changed microbiome in the latter. *Clostridium* is believed to induce gut inflammation and disrupt the intestinal epithelial barrier, thereby further promoting colonization by non-commensal pathogens [28]. The changes in the microbiota of 3- to 6-month-old infants have been shown to not only rely on feeding mode and delivery type but also depend on some other independent factors such as race/ethnicity and the cord blood vitamin D level. As an example, Caucasian infants have a less diverse microbiome but more *Bacteroides* in comparison to African American, while cesarean section causes an increase in diversity but decreases in *Bacteroides*, and formula-fed infants have increased levels of *Clostridium* [15]. However, in another study, the mode of delivery did not affect the diversity or the level of *Bifidobacteria*, but there were some differences in the abundance of the phyla Bacteroidetes and Verrucomicrobia, in the genera *Bacteroides*, *Akkermansia,* and *Kluyvera*, and in the species *B. longum*. In this specific study, only mothers with the Se+ phenotype were included, which could impact the results. Se+ means that they had an active FUT2 enzyme and produced high amounts of α1-2 fucosylated HMO (human oligosaccharides), such as 2′FL and lacto-N-fucopentaose I (LNFP I) in breast milk. This biocomponent is believed to be beneficial for the proper development of the infant microbiota [29].

HMOs are oligosaccharides with individual diversity and composition. So far, 200 types are known. They function not only as prebiotics but also impact epithelial barrier function, serving as a decoy to block the attachment of bacteria. A decrease in specifically one type, i.e., LNFPIII, has been indicated to be linked to cow milk food allergy in infants fed by mothers with low amounts of this HMO in breast milk [30]. To make the case more complicated, the introduction of solid food is an independent factor changing microbiota diversity in breastfed infants. The early life gut microbiota become more diverse when allergenic foods are introduced and mature toward a *Bacteroides*-rich community at the age of 12 months. Significant changes have been observed at a younger age in infants with early introduction of allergenic solids, beginning from 3 months of life [23]. It appears that this kind of intervention has the potential to change the distinct characteristics of the breastfed and formula-fed microbiome [31].

Another factor that could influence the infant’s intestinal microbiome is maternal metabolic status. Since there is a link connecting maternal obesity to allergy in offspring [32], this factor seems to affect the child’s gut microbiome primarily, both in utero and at birth, resulting later in dysbiosis and the development of unfavorable outcomes such as obesity [33,34] or allergy [35]. Maternal obesity also modifies breast milk composition resulting in low n-3 and elevated n-6 PUFA levels, with its further consequences [36].

In summary, breastfed infants present with lower diversity of the gut microbiota, breastfeeding (BF) is a strong predictor of the gut microbiota, and the cessation of BF is associated with a shift toward an adult-type microbiota. The predominant genus in the gut of BF infants is *Bifidobacterium*, with less abundance reported for *Firmicutes* and *Bacteroides* [37].

## 5. Supplements and Breastfeeding

Some supplements have been shown recently as possible modifiers of BF effect, with the main impact on microbial composition. The introduction of the supplement EVC001 (*Bifidobacterium* *infantis*), which utilizes HMO, a biocomponent of human milk that cannot be digested but act as a nutrient for bacteria only, was found to switch the immune response with a decrease in pro-allergy Th2 and pro-inflammatory Th17 response and an increase in INF-β. These events indicate the induction of tolerance to the intestinal microbiota, a crucial process for the healthy development of the immune system. *Bifidobacterium infantis* was discovered recently in the microbiota of infants from developing countries. This bacteria co-evolved with humans but is rare in the “modern” countries of Europe and North America. Studies performed in Sweden confirmed the absence of the gene for processing HMO in the bacteria metagenome profile of breastfed infants born in that country. *Bifidobacterium infantis* expresses all the genes necessary to utilize HMO [21]. In addition, HMO added to formula starting at 0–14 days and continuing up to 6 months had the effect of fewer infections and more *Bifidobacteria* in the gut, an effect similar to that of breastfeeding [38]. Similarly, *Lactobacillus* *rhamnosus* supplementation in breastfed infants from birth up to the second year of life, together with supplementation in mothers from 35 weeks of gestation up to 6 months after birth or until the end of breastfeeding, decreased the risk of atopic dermatitis at 11 years, and a lifetime decrease in the prevalence of eczema, atopic sensitization and wheeze [20]; such supplementation during pregnancy only did not have such an effect [39]. Another intervention based on the introduction of the oligosaccharide FUT2 in breastfed infants showed a reduced risk of atopic dermatitis at 2 years old. FUT2 is genetically polymorphic in mothers and determines the breast milk glycan composition and the variation of specific human milk oligosaccharides, which act as prebiotics. Thus, this factor seemed to impact the microbiota composition, also explaining the differences between human milk from different subjects. Non-secretor mothers, who lack a functional FUT2 enzyme, characterize approximately 15–25% of mothers depending on ethnic background. The presence of FUT2-dependent oligosaccharides is associated with the establishment of a *Bifidobacteria*-loaded microbiota [19]. Probiotics consisting of *Bifidobacterium breve* Bb99 (Bp99 2 × 10^8^ cfu) *Propionibacterium freundenreichii* subsp. shermanii JS (2 × 10^9^ cfu), *Lactobacillus rhamnosus* Lc705 (5 × 10^9^ cfu), and *Lactobacillus rhamnosus* GG (5 × 10^9^ cfu) given to both mothers and infants have been shown to modify the risk associated with cesarean section and the use of antibiotics early in life, by impacting the microbiota up to the third month of life, but only in breastfed infants. Breastfed infants also showed the expected increase in *Bifidobacteria* and a reduction in *Proteobacteria* and *Clostridium* [17].

Supplementation with different types of probiotics is showing promising results as a method of prevention against allergy. However, more caution is necessary with the way how the microbiota is being changed, specifically if one single component is modified. Possibly more natural, diet-driven interventions will be studied in the future.

## 6. Introduction of Solid Foods

Another factor that plays a major role in the development of allergic phenotype is an allergenic foods introduction. The early or late exposure could change the direction of immune events, inducing sensitization or tolerance. Additionally, BF seems to be impacting this specific effect. Allergenic foods play different roles depending on the timing of introduction but also the interaction of the intervention in both lactating mothers and children. Only the introduction of peanuts to both the mother during lactation and the child before 12 months of life resulted in a reduction in peanut allergy at 7 years old. Peanut antigens, given through breast milk, are distributed to the infant together with multiple bioactive factors, including maternal immunoglobulins, cytokines, microbiota, and immune cells, which possibly prime the infant’s immune system to develop tolerance when peanut is consumed a few months later by the child [22].

Since the 1990s, national societies have recommended delaying the introduction of common allergenic foods, including peanuts, until 2 or 3 years of age. However, despite these recommendations, the prevalence of food allergy increased over the following decades, leading to skepticism regarding delayed introduction as an effective prevention measure. Recent studies mentioned earlier in this review suggest that early introduction of allergenic food may rather reduce the risk of developing food allergy. In addition, studies in animal models have shown that maternal milk factors such as TGF-β, vitamin A and maternal OVA-specific IgG are required for the induction of oral tolerance when OVA is transmitted through the breast milk [40,41].

In contrast, the presence of aeroallergens in breast milk seemed to have the opposite effect in the case of dust mite allergens in a mouse model. Most inhaled proteins are likely ingested due to respiratory tract mucociliary clearance and go the same way in breast milk as food antigens. This theory was confirmed by finding the presence of *D*. *pteronyssinus* allergen in human digestive fluids. The presence of *D. pteronyssinus* in human milk has been shown to be associated with allergic sensitization, allergic rhinitis, and asthma in children [42]. *D. pteronyssinus* in breast milk seems to have the effect of priming the allergic response in adulthood, both in mice and humans and may interfere with the induction of oral tolerance to other food antigens. In a mouse model, *D.* *pteronyssinus* increased epithelial permeability, IL-33 expression, as well as ILC2 and Th2 differentiation while blocking the formation of Treg, processes related to allergic reactions [43].

The significance of allergen presence in breast milk and the early introduction of allergenic foods to infant diet for allergy development stays controversial for decades. It is not clear the way some allergens seem to be inducing tolerance when others have the opposite effect. The explanation emerging from discussed studies points to the immune status of lactating mothers as a key player.

## 7. Mechanism of the Effect of Breastfeeding

As epigenetics translates the environmental influence on genetic risk, feeding mode has been shown to impact methylation, with stable changes up to 10 years of life and a lowered global methylation profile in formula-fed infants. One study analyzed the methylome that formed on the Isle of Wight in children born in 1989 and 1990. In total, 87 CpGs were associated with the feeding mode, with 27 distinctly related to exclusive breastfeeding. The described effect could be caused by the bio-compounds present in the human milk and indirectly caused by changes in the microbiota, with known SCFAs (short-chain fatty acids) role as epigenetic modifiers [24]. Another study revealed changes in the methylation of DMs (differentially methylated site) at SNH25, which is related to the regulation of TGF-β and further production of IgA, and DMR (differentially methylated region) at FDFT1, related to hyperlipidemia, as a marker of breastfeeding duration and methylation at 10 years of age [44]. An EWAS (epigenetic wide association study) study on the ALSPAC (Avon Longitudinal Study of Parents And Children) cohort assessed the long-term effect of breastfeeding but revealed differences in methylation at only two DM sites and 12 DMRs related to breastfeeding (markers present at 7 and 15–17 years but not at birth) with a small global effect and absent dose-response relationship [45].

Nevertheless, within the same cohort, another study showed that DNA methylation was associated with 3 to 5 months of exclusive breastfeeding and slower BMI increase in the first 6 years of life in a dose-response manner with exclusive breastfeeding duration [46]. Another study identified six novel CpG sites associated with breastfeeding duration using an EWAS approach. One DM presented consistent associations with breastfeeding (cg00574958, CPT1A) in infancy and childhood but not at birth, while two differentially methylated sites in infancy (cg19693031, TXNIP; cg23307264, KHSRP) were not present at birth but did not persist into childhood [47]. In the past, some candidate gene studies have been evaluated with significant results for NPY, LEP, and Slc2a4 [48].

**Figure 1:**
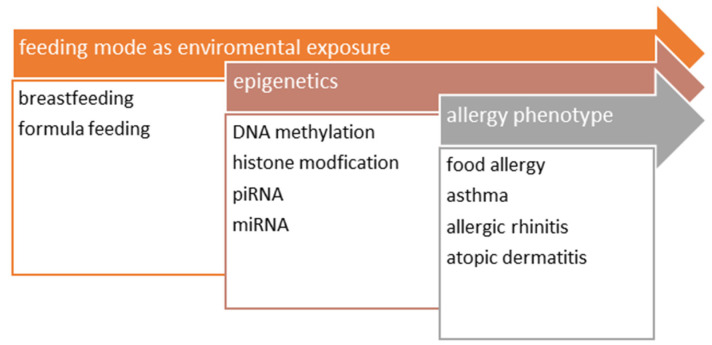
The figure illustrates the epigenetic effect of BF.

Other epigenetic mechanisms apart from DNA methylation have been taken into account, such as histone modifications, known for the possible epigenetic effect of dietary PUFA (polyunsaturated fatty acids) and the expression of PRKC2, FOXP3, IL10RA, and IL7R [49]. Moreover, epigenetic modifications are believed to be transgenerational via piRNA and miRNA. For the latter, there are interesting findings in animal studies indicating epigenetic modifications by the abnormality rates in milk siblings [50]. These may have further consequences in terms of milk biobanking. Although nursing by adoptive mothers has been performed for centuries, the exact consequences of such an action have never been considered. Breast milk seems to be infant-specific, and the composition is modified by both fetus-mother and later infant-mother interplay [51,52]. Even though pasteurization of donor milk is mandatory in order to destroy high-risk viruses and non-spore-forming bacteria, studies investigating the HoP (Holder pasteurization) process (62.5 for 30 min) reported conflicting results, with one reporting no change in selected miRNAs and the other showing substantial degradation [53]. Some important miRNA affecting different developmental properties in a child are *miR-148a-3p*, *miR-182-5p*, *let 7f-5p*, *miR-21* and *let7-c* [51]. The scheme presenting the effect of BF on allergy phenotypes is presented in Figure 1.

The epigenetic effect relies on the direct action of breast milk bio-compounds such as PUFA, miRNA, and piRNA, but also on indirect effects dependable on the microbiota composition. Both PUFA and microbiome variations provide some opportunity for future interventions.

## 8. Breastfeeding in Atopic Mothers

A possible confounder considering BF effect could be maternal atopy. Atopic mothers have less SCFA in their milk, which has been shown to be responsible for less protective effects in terms of allergy. SCFAs (formate, acetate, propionate, butyrate, and valerate) are interim and final products of alimentary carbohydrate fermentation by gut bacteria [25]. The controversy around breast milk from atopic mothers was indicted before, but only one large study showed that exclusive breastfeeding by atopic mothers for 3 months was associated with an increased risk of asthma at 7 years of life. The reverse relationship may exist before this time point, as shown in a study conducted in Tasmania estimating the risk of having asthma in relation to the mode of feeding in subjects 14–44 years old [54]. In another study, among children with a parental history of atopy (more maternal than paternal), breastfeeding added an additional risk of having allergic outcomes later in life. This association was further modified by the sex of children [55]. This finding is consistent with the speculation that the milk of mothers with atopy or asthma may differ with regard to immunologically active substances. It has been shown that the maternal IgE level is associated with IgE in the child only if the child is breastfed, and further, that prolonged breastfeeding among children whose mothers had high IgE was associated with high IgE in the child. This was further confirmed by observations in mice born to non-allergic mothers that were then breastfed by asthmatic foster mothers, which later developed increased airway hyperresponsiveness and eosinophilic airway inflammation [56]. However, somehow contrary to previous results in established risk/protection factors related to BF, this mode of feeding and vaginal delivery seems to modify the risk connected to maternal atopy [57] and asthma [58,59].

Another factor largely modifying the risk of allergic sensitization in children is the presence of allergen-specific IgG derived from mothers during pregnancy and lactation. It has been shown that the levels of these immunoglobulins are comparable in different sources, e.g., cord blood, the serum of mothers, and breast milk. The higher the concentration of allergen-specific IgG, the lowest the risk of IgE sensitization to the same allergen. Even though these levels could vary according to maternal allergy status, a protective effect has been shown to be present for both sensitized and non-sensitized mothers [60].

Based on the discussed studies, it is reasonable to say that atopy in pregnant and lactating mothers could modify the definitive effect of BF on allergy development in children, depending on the SCFA concentration and the immune status of mothers.

## 9. Conclusions

Breastfeeding is still the most recommended mode of feeding in infancy for its benefits to general health. However, with regard to allergy risk, the results of population-based studies in the last years are still inconclusive. According to the new research in that matter, based mainly on clinical trials, the effect could be modified by different genetic (HMO composition), environmental (cesarean section, allergen exposure), dietary (SCFA, introduction of solid food), and immunologic factors (IgG, IgE), thus explaining partially the differences observed in the population studies.

## Figures and Tables

**Table 1:** Summary of the recent literature regarding breastfeeding (BF) and allergy outcomes.

	Intervention or Observation	Age of Intervention or Observation	Type of Study	Outcome	Age of Outcome	Number of Participants	Effect of BF on Outcome	Limitations	Conclusion
	Cow milk exposure								
Urashima M, 2019 [9]	Avoiding supplementation with cow milk	1 day–5 months	RCT	Sensitization to cow milk Food allergy (including CMA and anaphylaxis)	2 years	312	RR 0.52 (0.34–0.81)	Amino acid formula in avoiding CM arm and switching to CM arm after 3 days	Sensitization to cow milk is preventable by avoiding CMF for at least 3 days of life
Sakihara T, 2021 [10]	Early introduction and daily infant CMF	1–2 months	RCT	CMA by OFC	6 months	504	RR 0.12 (0.01–0.5)	Soya-based formula in no CMF arm	Daily ingestion of CMF prevents CMA development
	BF effect as only exposure								
Ek WE, 2018 [11]	BF yes or not	time of BF	Cohort Born 1937–1969	Self-reported asthma hay fever eczema	38–73 years	336,364	Asthma OR 0.99 (0.96–1.02) hay fever/eczema OR 1.06 (1.03–1.08)	Wide time interval, population with different environmental exposure and cultural behaviors	BF is associated with an increased risk for hay fever and eczema, no effect on asthma
Flohr C, 2018 [12]	BF promotion	birth	Cluster RT	Spirometry Eczema Asthma Wheezing	16 years	17,046	Eczema OR 0.46 (0.25–0.83)	Allocation was not blinded	BF reduces eczema risk but not asthma
Filipiak-Pirttroff B, 2018 [13]	Exclusive BF for 4 month or supplementation with randomized formula Non-intervention group—no recommendations	birth	RCT	Asthma Eczema Allergic rhinitis	1, 2, 3, 4, 6, 10, and 15 years	5991	non-risk non-intervention allergic rhinitis OR 0.65 (0.42–0.99)	Recall bias in non-intervention group	In the non-intervention non-risk cohort—BF showed no effect on eczema and asthma, but a risk reduction for allergic rhinitis
Hu Y, 2021 [14]	Duration of BF	6–11 years	Population based	Asthma Allergic rhinitis Urticaria Food allergy Drug allergy	6–11 years	10,464	Asthma (and vaginal delivery) OR 0.78 (0.66–0.92)	Self-reported allergy Recall bias	BF > 6 months is inversely associated with childhood asthma and allergic diseases and modifies the risks of parental allergy and Cesarean section
	BF and microbiome composition								
Sordillo JE, 2017 [15]	Infant gut microbiome VDAART study (supplementation with low and high vitamin D at pregnancy) High-risk infants (atopic mother or father)	Pregnancy—vitamin D Infancy BF	RCT	Gut microbiome composition	3–6 months stool	333	beta −0.45 *p* < 0.001	High-risk infants, No allergy phenotype was studied at that point	Ethnicity, mode of delivery, BF, and cord blood vitamin D levels are associated with infant gut microbiome composition
Savage JH, 2018 [16]	Intestinal microbiome in breastfed high-risk infants (atopic mother or father) VDAART study (supplementation with low and high vitamin D at pregnancy)	pregnancy	RCT	Microbial composition	3–6 months	323	Bifidobacterium beta 0.56 (0.12, 1.00) Lactobacillus beta 3.50 (2.14, 4.86)	Included only high-risk infants	BF is dietary factor independently associated with microbiome composition
Korpela K, 2018 [17]	Probiotic supplementation with BF High-risk infants	pregnancy and infancy until 6 month	RCT	Intestinal microbiota composition	3 months	428	NA	Studying microbiota only, not proving any impact on allergy risk	At least partial breastfeeding together with probiotic supplementation might correct unfavorable changes in microbiota composition (possibly related to allergy risk) caused by antibiotics and cesarean birth
Lee-Sarwar KA, 2019 [18]	Intestinal microbiome VDAART study	pregnancy	RCT	Asthma at 3 y	3 years	361	beta 0.02 (0.01–0.03)	Parent reported asthma Not all metabolites were included Only high-risk children	Asthma-associated intestinal metabolites are significant mediators of the inverse relationship between exclusive breastfeeding for the first 4 months of life and asthma
	Supplement use with BF								
Sprenger N, 2017 [19]	FUT2-HMO measurement in the placebo group from supplementation with probiotics and prebiotics trial high-risk infants	Mean 2.6 day	RCT	Allergy IgE-allergy Eczema IgE-eczema	2 years 5 years	266	beta −2.14 SE 1.23 *p* = 0.083	High-risk infants Trend only	A lower risk of manifesting IgE-associated eczema at 2 years, but not 5 years, when fed breast milk with FUT2-HMO
Wickens K, 2018 [20]	Supplementation with either Lactobacillus rhamnosus HN001 *Lactobacillus rhamnosus* HN001 or *Bifidobacterium lactis* HN019	Mothers from 35 weeks of pregnancy—6 month Postpartum; children 1 day–2 year	RCT	Eczema Asthma Wheeze Rhinitis	10 years	298	12 months prevalence eczema RR 0.46 (0.25–0.86) hay fever RR = 0.73 (0.53–1.00) Lifelong prevalence atopic sensitization HR = 0.71 (0.51–1.00) eczema HR = 0.58 (0.41–0.82) wheeze HR = 0.76 (0.57–0.99)	Study not directed at BF, mixed effect of maternal and child’s diet supplementation	HN001 supplementation is associated with a significant reduction in hay fever, eczema, wheeze, and atopic sensitization
Henrick BM, 2021 [21]	Supplementation with B.infantis EVC001 Metagenomics profiling of BF infants	7–28 day *n* = 60	CT	Metagenomics profile *n* = 288 Galectin-1 Th2 Th17 *n* = 60	1–6 month	208 Sweden 60 U.S.	NA	No intestinal tissue studied	Infants colonized early in life with Bifidobacterium species are less likely to develop immune-mediated diseases
	Solid food introduction								
Pitt TJ, 2018 [22]	Peanut introduction before 12 month	Infancy and time of BF	Cohort	Peanut sensitization	7 years	545	OR 0.08 (0.01–0.85)	No data on environmental peanut exposure and peanut exposure during pregnancy	Maternal peanut consumption while breastfeeding paired with direct introduction is associated with a lower risk of peanut sensitization
Marrs T, 2021 [23]	Solid food regular consumption of 6 allergenic foods from 3 months alongside continued BF or EBF until 6 month	3 months	RCT	Intestinal microbiota Allergen-specific IgE Atopic dermatitis	6 months 12 months	288	NA	No data before 3 month	Introduction of allergenic solids from age 3 months alongside breastfeeding is associated with maturation of the gut microbiota
	Epigenetic effect of BF								
Mallisetty Y, 2020 [24]	Epigenetics of BF	Time of BF	Cohort IOWBC	Methylation in blood Lung function Serum IgE	birth 10 years 18 years	201	NA	Relatively small sample size	87 CpGs were identified as DM, the methylation pattern in EFF group was more stable from birth to 10 years and significantly lower cg25458520 (MAPK13 gene) is related to an increase in FEV1/FVC in EBF
	Atopic mothers								
Stinson LF, 2020 [25]	SCFA composition measurement in BM from atopic and non-atopic mothers	1 month	Cohort	SCFA composition	1 month	109	NA	No allergy phenotype in children studied	Atopic mothers had significantly lower concentrations of acetate and butyrate than non-atopic mothers

The table contains data from clinical trials (CT), randomized clinical trials (RCT), and population-based (cohort) studies. RR—relative risk, OR—odds ratio, beta—estimate in the regression model, BM—breast milk, CMF—cow milk formula, CM—cow milk, CMA—cow milk allergy, OFC—oral food challenge, SCFA—short fatty chain acids, EBF—exclusively breastfed, EFF—exclusively formula fed, VDAART—Vitamin D Atenatal Asthma Reduction Trial, FUT2—Fucosyltransferase 2 gene, IOWBC—The Isle Of Wight Whole Population Birth Cohort, HMO—human milk oligosaccharides, DM—differentially methylated NA—not applicable.

## Data Availability

Not applicable.

## Abbreviations

BF	breastfeeding
SCFA	short-chain fatty acids
HMO	human oligosaccharide
OVA	ovalbumin
ILC2	innate lymphoid cell type 2
